# Genetic risk analysis of a patient with fulminant autoimmune type 1 diabetes mellitus secondary to combination ipilimumab and nivolumab immunotherapy

**DOI:** 10.1186/s40425-016-0196-z

**Published:** 2016-12-20

**Authors:** Jared R. Lowe, Daniel J. Perry, April K. S. Salama, Clayton E. Mathews, Larry G. Moss, Brent A. Hanks

**Affiliations:** 1 Department of Medicine, Duke University Medical Center, Durham, NC 27710 USA; 2 Department of Pathology, Immunology, and Laboratory Medicine, University of Florida College of Medicine, Gainesville, FL 32610 USA; 3 Department of Medicine, Division of Medical Oncology, Melanoma Program, Duke Cancer Institute, Duke University Medical Center, Durham, NC 27710 USA; 4 Department of Medicine, Division of Endocrinology, Metabolism, & Nutrition, Duke University Medical Center, Duke Molecular Physiology Institute and Sarah W. Stedman Nutrition and Metabolism Center, Durham, NC 27701 USA

**Keywords:** Nivolumab, Ipilimumab, Type I diabetes, Advanced melanoma, Autoimmune endocrinopathy, Single nucleotide polymorphism, HLA risk allele, Genetic risk analysis

## Abstract

**Background:**

Checkpoint inhibitor immunotherapy is becoming an effective treatment modality for an increasing number of malignancies. As a result, autoinflammatory side-effects are also being observed more commonly in the clinic. We are currently unable to predict which patients will develop more severe toxicities associated with these treatment regimens.

**Case presentation:**

We present a patient with stage IV melanoma that developed rapid onset autoimmune type 1 diabetes (T1D) in response to combination ipilimumab and nivolumab immunotherapy. At the time of the patient’s presentation with diabetes ketoacidosis, a confirmed anti-GAD antibody seroconversion was noted. Longer-term follow-up of this patient has demonstrated a durable complete response based on PET CT imaging along with a persistently undetectable C-peptide level. Single nucleotide polymorphism gene sequencing and HLA risk allele analysis has revealed the patient to lack any established genetic predisposition to the development of autoimmune T1D.

**Conclusions:**

While larger studies are necessary to better understand the role of genetic risk factors for the development of autoimmune toxicities in those patients undergoing checkpoint inhibitor immunotherapy, these results suggest that pre-screening patients for known T1D risk alleles may not be indicated. Additional investigation is needed to determine whether an approach such as T cell receptor clonotypic analysis to identify the presence of autoreactive T cell clones may be an effective approach for predicting which patients are at risk for the development of autoinflammatory toxicities while undergoing checkpoint inhibitor immunotherapy.

## Background

Recent developments in cancer immunotherapy have led to the widespread use of immune checkpoint inhibitors for the management of advanced melanoma. This includes ipilimumab, a human IgG1 monoclonal antibody that blocks the negative checkpoint regulator CTLA-4 on the surface of activated T cells [1]. Additional checkpoint inhibitors designed to counteract the ability of tumor cells to dampen the host immune response include nivolumab and pembrolizumab, both IgG4 monoclonal antibodies that target the negative regulatory T cell surface receptor, PD-1 [2]. Each of these agents have been shown to significantly prolong overall survival in patients with advanced melanoma in phase III clinical trials [3, 4] and, in the case of the anti-PD-1 antibodies, are now being utilized in the treatment of other solid tumors reflected by the recent FDA approvals for their use in non-small cell lung cancer, renal cell carcinoma, and head and neck squamous cell carcinoma [5–7].

Early in their development, it was recognized that these immune checkpoint inhibitors were associated with a novel syndrome of autoimmune or autoinflammatory side effects [8]. These toxicities have become known as immune-related adverse events (irAEs) and occur more frequently when these agents are administered in combination regimens. The incidence of grade 3/4 irAEs has been reported in up to 23% of patients receiving ipilimumab, 14% of patients receiving nivolumab, and 54% of patients undergoing treatment with the ipilimumab and nivolumab combination regimen [3]. While several autoimmune conditions have been reported in patients undergoing these therapies, the most common irAEs include dermatitis, enterocolitis, hepatitis, as well as various endocrinopathies [2]. Depending on the pathway targeted, the more common endocrinopathies include autoimmune hypothyroidism often following a period of hyperthyroidism in the setting of thyroiditis, as well as autoimmune lymphocytic hypophysitis, often manifesting as anterior panhypopituitarism. Ipilimumab monotherapy at a dose of 3 mg/kg has been associated with the condition of hypophysitis in 3-9% of cases while the reported observed incidence increases to 12% in patients undergoing combinatorial checkpoint immunotherapy [2, 9].

Although relatively uncommon, cases of autoimmune diabetes, known as type 1 diabetes (T1D), have emerged in association with use of the anti-PD1 antibody therapies [10–15]. Herein, we report a case of fulminant T1D manifesting as diabetic ketoacidosis concurrent with autoimmune thyroiditis and acute adrenal insufficiency occurring in a patient with advanced melanoma undergoing combination nivolumab and ipilimumab immunotherapy. To better understand the development of T1D in the setting of combination anti-PD-1 and anti-CTLA-4 antibody therapy, we conducted a genetic risk analysis of this patient based on single nucleotide polymorphism (SNP) and HLA risk allele sequencing. We will review this experience in the context of other recent reports of immune checkpoint inhibitor-induced T1D and discuss the implications of these findings in terms of our understanding of the pathophysiology of T1D.

## Methods

### Patient

Treatment-related side-effects were graded according to the National Cancer Institute Common Terminology Criteria for Adverse Events (CTCAE), version 4.0.

### SNP analysis and GRS calculation

The patient’s PBMC-derived DNA sample was interrogated at the University of Florida Diabetes Institute for 26 unique SNP loci found to be associated with the pathogenesis of T1D according to ImmunoBase (https://www.immunobase.org/). The genetic risk score (GRS) was calculated as a sum of each risk allele multiplied by its associated ln(Odds Ratio (OR)) divided by the total number of risk alleles tested producing a range from 0 (no susceptibility alleles) to 1 (all susceptibility alleles) [16].

## Case presentation

A 54 year-old male with no significant past medical history was initially diagnosed with a cutaneous melanoma involving his left forearm in September 2012. He underwent local resection and sentinel lymph node biopsy at an outside institution with pathology showing an invasive melanoma characterized by an ulcerated Clark level V lesion with a Breslow thickness of 5.5 mm and 3 mitoses/mm^2^. A sentinel lymph node biopsy was positive for 1 of 1 lymph node with a 0.35 mm deposit of melanoma. A complete lymph node dissection of the left axilla was performed demonstrating no evidence of melanoma in any of 42 lymph nodes. He underwent surveillance imaging by whole body PET CT through June of 2013 with no evidence of disease recurrence.

In September of 2013, the patient noted development of a subcutaneous nodule in his left upper extremity. On examination, he was found to have unilateral swelling of his left lower extremity and a palpable intra-muscular mass. Fine-needle aspiration of the left upper extremity nodule was performed and pathology was consistent with melanoma. PET CT imaging performed in October of 2013 demonstrated a subcentimeter FDG avid lesion in the left upper extremity correlating with the subcutaneous nodule, as well as an intramuscular lesion in the left gastrocnemius with marked FDG uptake and a focal area of FDG uptake in the small intestine without a CT correlate.

**Fig. 1:**
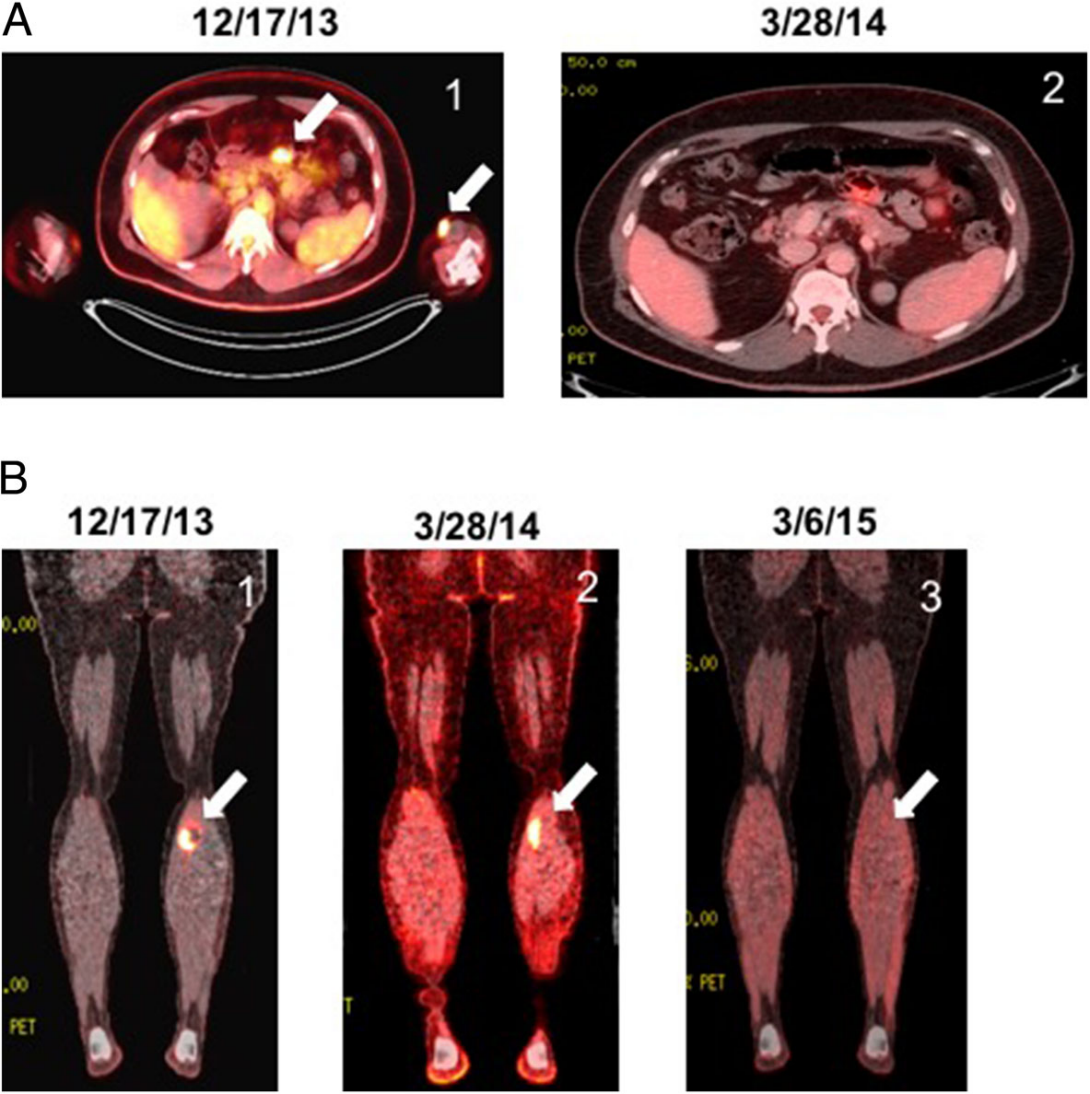
PET CT Imaging of Patient Undergoing Combination Ipilimumab/Nivolumab Immunotherapy. **a** *left arrow*, mesenteric lesion. *right arrow*, left upper extremity cutaneous lesion (1) prior to initiating combination ipilimumab/nivolumab therapy, (2) following the second dose of combination ipilimumab/nivolumab therapy. **b** (1) prior to initiating combination ipilimumab/nivolumab therapy, (2) following the second dose of combination ipilimumab/nivolumab therapy, (3) 1 year following the third dose of combination ipilimumab/nivolumab therapy and treatment discontinuation due to toxicity

**Fig. 2:**
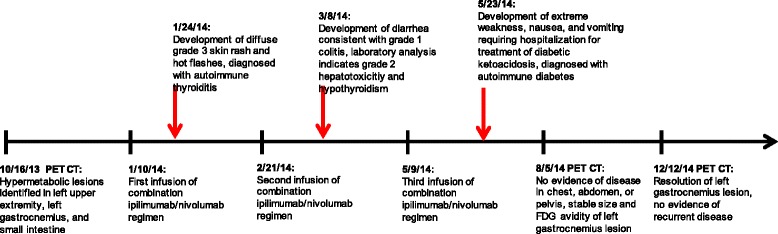
Time line of clinical events

The patient was referred to our melanoma clinic for further care in December of 2013. Repeat imaging with PET CT performed at that time confirmed prior FDG avid lesions involving the upper and lower extremities along with the small bowel lesion (Fig. 1). Brain MRI was negative. Molecular diagnostics performed on a tissue sample identified the melanoma as BRAF and c-kit wild type. The patient elected to enroll in the CheckMate-069 randomized, double blind clinical trial evaluating combination ipilimumab and nivolumab therapy versus ipilimumab monotherapy in previously untreated patients with advanced melanoma (NCT01927419). He received his first infusion in January of 2014, returning 2 weeks later with a diffuse grade 3 skin rash, tachycardia, and reports of hot flashes (Fig. 2). Laboratory analysis indicated elevated levels of free triiodothyronine at 9.68 pg/mL (normal 2.20 – 3.80 pg/mL) and free thyroxine at 4.16 ng/dL (normal 0.52 – 1.21 ng/dL) in the setting of a decreased thyroid stimulating hormone (TSH) level of 0.06 uIU/mL (normal 0.34 – 5.66 uIU/mL). Anti-microsomal antibodies were positive and his thyrotropin receptor antibody level was noted to be elevated at 4.74 IU/L (normal 0.00 – 1.75 IU/L). Autoimmune thyroiditis was diagnosed prompting treatment with prednisone and metoprolol while the rash was managed with topical steroids.

Once his symptoms resolved and he was tapered off systemic steroids, the second infusion was administered approximately 6 weeks later. Three weeks following his second dose of combination ipilimumab/nivolumab therapy, routine laboratory analysis indicated a grade 2 hepatotoxicity, attributed to treatment-induced autoimmune hepatitis, as well as symptoms consistent with a grade 1 colitis. Testing for CMV reactivation was not performed. He was treated with prednisone which was tapered as the transaminitis resolved. At this time the patient was found to have abnormally diminished levels of free triiodothyronine at 1.69 pg/mL and free levothyroxine at <0.25 ng/dL along with an elevated TSH at 7.50 uIU/mL. Given his hypothyroid state, his metoprolol was stopped and therapy with levothyroxine was initiated.

Repeat imaging with PET CT in March of 2014 showed complete resolution of the left upper extremity nodule and small intestine lesions and decreased size and FDG avidity of the left gastrocnemius lesion (Fig. 1). Diffuse colitis was apparent on imaging. The patient continued to report one to two loose stools a day throughout his treatment course and his symptoms were managed with loperamide in addition to the previous prednisone taper.

In May of 2014 the patient received his third infusion. Two weeks later, he returned to the clinic with extreme weakness, myalgias, nausea, and vomiting. His vitals showed a blood pressure of 88/54 mmHg with a pulse of 121, and his laboratory analysis revealed profound hyperglycemia with an anion gap metabolic acidosis. Due to concerns for new onset diabetic ketoacidosis with acute adrenal insufficiency, he was transferred directly to the medical intensive care unit. Intravenous fluids, insulin, and methylprednisolone were initiated. His β-hydroxybutyrate level was noted to be elevated at 0.40 mmol/L while his ACTH was found to be undetectable and his cortisol level was 3.5 μg/dL (normal 5.0 – 25.0 μg/dL). The patient’s C-peptide was <0.1 ng/mL, and he was noted to have elevated levels of anti-glutamic acid decarboxylase (anti-GAD) antibodies during his hospitalization at 0.38 nmol/L (normal ≤0.02 nmol/L). The patient was eventually transitioned from intravenous insulin to a subcutaneous regimen, steroids were tapered to hydrocortisone, and he was discharged to home after a 3-day admission. Notably, further studies on serum previously collected per study protocol showed that the patient exhibited an undetectable anti-GAD antibody titer 1 month prior to initiating treatment with combination ipilimumab/nivolumab immunotherapy.

As the patient had demonstrated evidence of multiple endocrinopathies, further evaluation of his pituitary function was conducted. This included a prolactin of 6.51 ng/mL (normal 2.64 – 13.13 ng/mL), LH of 3.8 mIU/mL (normal 1.4 – 7.7 mIU/mL), FSH of 17.8 mIU/mL (normal 1.3 – 19.3 mIU/mL), free thyroxine of 0.84 ng/dL (normal 0.52 – 1.21 ng/dL), testosterone of 64 ng/dL (normal > 300 ng/dL), and free testosterone of 1.9 ng/dL (normal > 9 ng/dL). A subsequent brain MRI performed with pituitary protocol was unremarkable and unchanged compared with a baseline study. An early morning Cosyntropin stimulation test was conducted in October of 2014 following a 5 month maintenance dosage of prednisone at 10 mg daily which was tapered to discontinuation over a period of 6 weeks prior to stimulation testing. Baseline testing of ACTH and cortisol remained undetectable while the cortisol level post-stimulation was inappropriately low at 1.8 microgram/dL (normal 5.0 – 25.0 microgram/dL). Together, these findings were felt to be consistent with hypophysitis.

In light of the multiple autoinflammatory toxicities, the patient was removed from the study and placed on surveillance comprised of a physical exam, lab assessment, and PET CT imaging every 3 months in addition to dermatologic surveillance every 6 months. Soon thereafter, it was revealed that the patient had been randomized to the combination ipilimumab and nivolumab immunotherapy regimen. His follow-up exams continued to reveal a stable 1.1 cm nodule with low-level FDG avidity within the left gastrocnemius muscle with no other evidence of disease recurrence until September of 2014 when the left gastrocnemius lesion was no longer FDG avid. As of August 2016, the patient has been without evidence of melanoma recurrence and continues on an insulin regimen with an undetectable C-peptide level.

## Conclusions

The exact mechanisms by which immune checkpoint inhibitors induce autoimmunity and cause irAEs are not fully understood. Recent research on anti-CTLA-4 associated hypophysitis suggests that local expression of CTLA-4 in the pituitary leads to antibody binding and activation of the complement pathway with subsequent site-specific inflammation and tissue injury [17]. However, this work is observational, and the exact pathophysiology of other irAEs, including the possible role of complement-mediated injury, remains elusive. It is suspected that checkpoint inhibitors interfere with peripheral tolerance and potentiate autoimmune disorders in genetically predisposed individuals [5]. While the occurrence of irAEs in an individual on combination checkpoint inhibitor therapy is expected, the dramatic presentation of fulminant T1D and its potential for other complications is notable and warrants further consideration.

Autoimmune diabetes is characterized by the development of an adaptive immune response against specific β-cell antigens [18]. Longitudinal studies in patients have shown that certain autoantibodies, such as anti-insulin (IAA), anti-islet cell antigen 512 (ICA512), and anti-glutamic acid decarboxylase (GAD65), define preclinical disease as they are present in the serum for years prior to symptom development [19]. They have been shown to be markers heralding a disease state but do not directly mediate disease progression. This is also true for patients with latent autoimmune diabetes mellitus in adults (LADA), which has a slow progression to insulin dependency that may be predicted by the presence of islet autoantibodies [20]. Certain cases of T1D are characterized as fulminant if the patient presents with diabetic ketoacidosis soon after the onset of hyperglycemic symptoms, has a near normal HbA1c, and has a low C-peptide level [21]. These patients typically do not have autoantibodies, the absence of which is thought to be indicative of the rapid nature of the disease pathogenesis [22]. In the case presented here, the concurrent development of both anti-GAD65 antibodies and insulin dependency is highly atypical. Indeed, to our knowledge, it is the first reported case of documented seroconversion temporally associated with immune checkpoint inhibitor therapy.

The clinical presentation of T1D is due to the lack of endogenous insulin secondary to the destruction of insulin-producing β-cells in the pancreas. This process is attributed to infiltration of the pancreatic islets by autoreactive T cells, causing ‘insulitis’ [23]. The most widely used model to study this process is the non-obese diabetic (NOD) mouse. In this population, insulitis and overt diabetes spontaneously develop at 20 to 30 weeks of age depending on gender. The NOD mouse model has been used to assess the relationship between disease progression and various alleles related to immune regulation, including the pathways targeted by checkpoint inhibitor therapies, PD-1 and CTLA-4 [24]. Studies in this murine model have implicated a cooperative relationship between these negative regulatory receptors in the pathogenesis of T1D via immune dysregulation [25, 26]. CTLA-4 has a role in modulating the survival and function of the regulatory T cell population, which is responsible for suppressing autoreactive T cell activation. The importance of CTLA-4 in regulating autoimmunity was demonstrated early on in CTLA-4 knockout mice, in which CD4-predominant lymphoproliferation develops and results in T cell infiltration of multiple organs and early mortality at 3–4 weeks [27]. Indeed, studies by Ansari et al. demonstrated that CTLA-4 blockade in NOD mice induces T1D. However, blockade of this pathway was noted to only induce diabetes in neonates and not in adult mice, suggesting that the role of CTLA-4 may be limited to inhibiting the activation of naïve T cells [26]. In contrast, PD-1 blockade precipitates diabetes in NOD mice at any age. This is likely due to PD-1 involvement in inhibiting both naïve T cells as well as the effector function of activated autoreactive T cells [26]. Another role of PD-1 in regulating peripheral tolerance for these autoreactive T cells is to modulate T cell mobility. Fife et al. demonstrated that blockade of PD-1 in pre-diabetic NOD mice inhibits T cell migration, thus promoting the formation of stable conjugates between T cells and antigen presenting dendritic cells by prolonging their interactions. The authors concluded that this results in increased T cell activation and precipitates diabetes in NOD mice [28]. The protective effect of PD-1 has also been suggested in humans as individuals with T1D have significantly lower levels of CD4^+^ T cell PD-1 expression compared to healthy controls [29]. Taken together, these studies indicate that the CTLA-4 and PD-1 pathways function at different stages in the development of T cell tolerance to prevent autoimmune diabetes [30].

This cooperative relationship has been further supported by studies demonstrating that the risk of T1D is directly related to the degree of genetic predisposition. Work by Kochupurakkal et al. in NOD mice demonstrated that specific strains are protected from developing T1D after PD-1 blockade by having functional alleles for *Il2* and *Ctla4*. It was speculated that the functional *Ctla4* allele with the help of IL-2 was sufficient to maintain self-tolerance and prevent T1D [25]. While the exact mechanism remains unclear, similar associations between the risk of developing T1D and variations in alleles for CTLA-4 and PD-1 have been identified in multiple human populations [31–35].

Another genetic predisposition for developing T1D, and perhaps the most widely studied, relates to the major histocompatibility complex (MHC) and the associated human leukocyte antigen (HLA) molecules. HLA class I and class II molecules are responsible for presenting endogenous and exogenous antigens, respectively, to T cells and initiating the immune response. Specific variations in HLA-I and HLA-II molecules are associated with increased risk of or protection against the development of T1D [36]. The incidence of T1D in association with checkpoint inhibitor therapy has been previously reported as part of a larger case series, and a few reports have included HLA typing of the affected individuals [10–15, 37]. Of the reported cases of immunotherapy-associated T1D included in Table 1, HLA typing was performed on eight individuals. Six of these individuals expressed the HLA-II DR4 haplotype, which is a well-known risk allele for T1D with an odds ratio (OR) of 5.68 for developing the disease [36, 38]. Our HLA typing of this patient identified the HLA-I A2 and HLA-II DQB1*0602 alleles with no evidence of a previously characterized HLA risk allele for T1D. HLA-II DQB1*0602 is associated with one of the most protective haplotypes for developing T1D with an OR of 0.03, though this protective effect seems to be restricted to childhood [38, 39]. We conclude that the HLA typing for this patient is not consistent with an immunologic predisposition to the development of T1D.

**Table 1:** Reported cases of immunotherapy-associated T1D

Study	No. of patients	Patients	Age/Sex	Past medical history	Checkpoint inhibitor therapy	Positive serologies	Genetics	Comments
Gaudy et al.	1	1	44/F	None	Pembrolizumab	Unavailable	Unavailable	
Martin Liberal et al.	1	1	54/F	Mild asthma	Ipilimumab and pembrolizumab	GAD65 70.1 U/mL	DRB1*04, DQB1*0302 (HLA A2 DRA DQ8)	
Hughes et al.	5	1	55/F	Autoimmune thyroid disease	Nivolumab	None	A2.1, DR4	Patient had previously progressed through ipilimumab
		2	83/F	Remote smoker	Nivolumab	GAD65 1.2 U/mL	A2.1, DR4	
		3	63/M	Hypertension	Nivolumab	GAD65 1.1 U/mL, ICA5 1.2 U/mL, IAA 47 U/mL	A2.1, DR4	
		4	58/M	Type 2 diabetes mellitus	Nivolumab	GAD65 13819 U/mL	A2.1	
		5	64/F	Autoimmune thyroid disease, psoriasis	Pembrolizumab	None	DR4	
Okamoto et al.	1	1	55/F	Dyslipidemia, gastric ucler	Nivolumab	None	DRB1*04:05, DQB1*04:01 (DR4)	
Miyoshi et al.	1	1	66/F	None	Nivolumab	None	DRB1*11:01 13:02:01, DQB1*03:01:01 06:04:01	
Brahmer et al.	1	1	unavailable	Unavailable	BMS-936559 anti-PDL1 antibody	Unavailable	Unavailable	
Hoffmann et al.	3	1	70/F	None	Nivolumab	None	Unavailable	Patient had previously progressed through ipilimumab
		2	78/F	Type 2 diabetes mellitus	Nivolumab	GAD positive	Unavailable	
		3	58/F	None	Pembrolizumab	GAD, IAA positive	Unavailable	

Diabetic autoantibodies referenced include GAD65, ICA5, and insulin (IAA). Normal GAD65 titers < 0.5 U/ml, ICA5 < 1.0 U/ml, IAA < 5.0 U/ml

In order to investigate this patient’s genetic risk for developing T1D in more detail, we also conducted a SNP analysis of 26 different loci previously associated with the development of T1D and calculated a genetic risk score (GRS) emulated from Oram et al. [16]. This GRS summarizes risk-associated variation across the genome. In this case, the patient’s GRS was 0.2072, a score that is below the 5^th^ percentile of the T1D cohort [16]. These findings indicate that this patient did not have a genetic profile consistent with any known predisposing factors for the development of T1D.

This case demonstrates that combination ipilimumab and nivolumab immunotherapy is capable of inducing the development of T1D even in those patients with no discernable risk factors for this disease. These findings highlight the importance of the PD-1 and CTLA-4 negative regulatory T cell receptors in the pathogenesis of T1D and suggest that dual checkpoint blockade may be unleashing the activation of previously existing islet-reactive T cell clones in healthy individuals. Given that all reported cases of checkpoint inhibitor-related T1D have been associated with anti-PD-1 antibody therapy (Table 1), it seems likely these islet-reactive T cell clones reside in or near pancreatic β-islet tissues or their associated draining lymph node tissues prior to therapy.

In summary, multiple studies have implicated both the CTLA-4 and PD-1 pathways in the pathogenesis of T1D and suggest a synergistic relationship between these two negative regulatory receptors to potentiate autoimmune disorders. Kochupurrakal et al. concluded that the combined blockade of the CTLA-4 and PD-1 pathways poses a risk of disrupting peripheral tolerance and generating T1D [25]. This hypothesis has been supported by studies of concurrent nivolumab and ipilimumab administration for the treatment of advanced melanoma, in which the incidence of grade 3 or 4 irAEs was higher than in trials of either agent alone [40]. While the HLA-II DR4 haplotype has been previously reported in cases of immunotherapy-associated T1D (Table 1), whether these risk alleles predict for the development of irAEs in patients undergoing checkpoint inhibitor immunotherapy remains unclear. Given the lack of association of any known genetic risk allele for T1D in the case presented here and the rarity of this complication in patients undergoing immunotherapy, the use of established genetic and immunologic screening studies for T1D prior to initiating a patient on checkpoint inhibitor immunotherapy may not be indicated. However, this represents a single case report and more definitive studies will be necessary to address this issue. Finally, this study suggests that additional investigation is needed to determine whether T cell receptor clonotypic analysis to identify the presence of either peripheral blood or tissue-resident autoreactive T cell clones may be an effective approach for predicting which patients are at increased risk for developing autoinflammatory toxicities while undergoing checkpoint inhibitor immunotherapy.

## Abbreviations

CTLA-4: Cytotoxic T-lymphocyte-associated antigen-4
FDG: Fluorodeoxyglucose
irAE: Immune-related adverse event
LADA: Latent autoimmune diabetes mellitus in adults
PD-1: Programmed death-1
PET: Positron emission tomography
T1D: Type I diabetes mellitus
T2D: Type II diabetes mellitus
